# New Epidemiological and Clinical Signatures of 18 Pathogens from Respiratory Tract Infections Based on a 5-Year Study

**DOI:** 10.1371/journal.pone.0138684

**Published:** 2015-09-25

**Authors:** Xiaohong Liao, Zhengbo Hu, Wenkuan Liu, Yan Lu, Dehui Chen, Meixin Chen, Shuyan Qiu, Zhiqi Zeng, Xingui Tian, Hong Cui, Rong Zhou

**Affiliations:** 1 State Key Laboratory of Respiratory Diseases, Guangzhou Medical University, Guangzhou, Guangdong, China; 2 The First Affiliated Hospital of Guangzhou Medical University, Guangzhou, Guangdong, China; 3 Department of Orthopedics, Zhujiang Hospital, Southern Medical University, Guangzhou, Guangdong, China; Kliniken der Stadt Köln gGmbH, GERMANY

## Abstract

**Background:**

Respiratory tract infections (RTIs) are a heavy burden on society. However, due to the complex etiology of RTIs, the clinical diagnosis, treatment, and prevention of these infections remain challenging, especially in developing countries.

**Methods:**

To determine the epidemiological and clinical characteristics of 18 respiratory pathogens, we analyzed 12,502 patients with acute respiratory infections (ARIs) by performing polymerase chain reaction (PCR) on patient pharyngeal swabs.

**Results:**

Samples positive for at least 1 pathogen were obtained from 48.42% of the total patients. Of these pathogen-positive patients, 17.99% were infected with more than 1 pathogen. Of the 18 pathogens analyzed, four were detected with a positive detection rate (PDR) > 5%: influenza A virus (IAV) > respiratory syncytial virus (RSV) >*Mycoplasma pneumoniae* (MP) > human coronavirus (HCoV). The pathogens with the 4 highest co-infection rates (CIRs) were as follows: HCoV > human bocavirus (HBoV) > enterovirus (EV) > parainfluenza virus (PIV). The overall positive detection rate (PDR) varied significantly according to patient age, the season and year of detection, and the disease subgroup, but not according to patient sex. The individual PDRs of the pathogens followed 3 types of distributions for patient sex, 4 types of distributions for patient age, 4 types of seasonal distributions, 2 types of seasonal epidemic trends, 4 types of yearly epidemic trends, and different susceptibility distributions in the disease subgroups. Additionally, the overall CIR showed significantly different distributions according to patient sex, patient age, and the disease subgroup, whereas the CIRs of individual pathogens suggested significant preference characteristics.

**Conclusion:**

IAV remains the most common pathogen among the pathogens analyzed. More effort should be directed toward the prevention and control of pathogens that show a trend of increasing incidence such as HCoV, human adenovirus (ADV), and RSV. Although clinically distinguishing specific pathogens responsible for RTIs is difficult, the epidemiological and clinical characteristics of the various RTI-causing agents could provide clues for clinicians, thereby informing decisions regarding prevention and medication and guiding appropriate public health strategies.

## Introduction

The etiology of respiratory tract infections (RTIs) is diverse and complicated. In the past decade, many new pathogens that cause RTIs have been identified, including human coronavirus (HCoV) [1], human metapneumovirus (HMPV) [2], and human bocavirus (HBoV) [3]. Advances in molecular techniques, have led to an increased detection rate for respiratory viruses, suggesting that the frequency of non-bacterial RTIs was previously underestimated. In 2008, the World Health Organization (WHO) reported that the annual worldwide incidence of RTIs was 156 million cases, of which 151 million occurred in developing countries, with 43 million cases occurring in India, 21 million cases occurring in Pakistan, and 10 million cases occurring in China. Additionally, 43% of RTI patients were reported to be infants or young children [4]. In 2011, the WHO estimated the worldwide incidence of RTIs to be 450 million cases, which were associated with approximately 4 million RTI-related deaths, accounting for 7% of all disease-related deaths [5]. Approximately 200 million of the RTI cases were caused by viral infections, and the positive detection rate (PDR) in pediatric patients was similar to that in adult patients. In developed countries, the predominant pathogens that cause RTIs are influenza A virus (IAV), influenza B virus(IBV), four types of parainfluenza viruses (PIV1, PIV2, PIV3, and PIV4), respiratory syncytial virus (RSV), adenovirus (ADV) and human rhinovirus (HRV) [6, 7]. In developed and developing countries, pediatric RTI cases in particular are mainly caused by RSV, HRV, HMPV, HBoV and PIV [8]. However, the characteristics associated with RTIs in developing countries have not been elucidated, and studies on the epidemiological and clinical signatures of RTIs are necessary. This study thus analyzed the incidences of 18 RTI pathogens in patients in Guangzhou, southern China, during the five-year period from July 2009 to June 2014. We also sought to identify the epidemiological and clinical characteristics of the RTI cases to better guide treatment and prevention strategies.

## Methods and Materials

Procedural details can be found in previous articles [9–11]. Briefly, this study was authorized by the ethics committee of the First Affiliated Hospital of Guangzhou Medical University, and all participants or their guardians provided written informed consent.

### Patients and collected specimens

We analyzed inpatients who presented to pediatric and respiratory departments from July 1, 2009, to June 30, 2014, and experienced at least 2 of the following respiratory symptoms or signs: cough; uncomfortable pharynx; stuffy nose; runny nose; sneezing; difficulty breathing; breathing distress; chest pain; asthma; and/or pneumonia or tracheitis, as indicated by X-ray, CT, or MRI. Pharyngeal swabs obtained from these patients were transported in viral media at 4°C to the State Key Laboratory of Respiratory Diseases and were tested either immediately or after being stored at -80℃ for no more than 72 hours. The data were divided into different groups according to the immunization capacity and national vaccination plan in China. Children who were ≤ 14-year-old were categorized into three subgroups (0–1, 2–6, and 7–14 years), and individuals > 14-year old was subdivided into three subgroups (15–30, 31–50, and ≥ 51 years). The percentages of individuals in each of these age subgroups were 28.72%, 32.51%, 6.90%, 13.07%, 9.07%, and 9.7%, respectively.

### Definition of groups by clinical diagnosis

The single acute upper RTI group (SAURTIG) consisted of patients with the following symptoms or signs: rhinitis, stuffy nose, runny nose, pharynx congestion, sore throat, tonsil inflammation, and/or cough.

The single acute lower RTI group (SALRTIG) consisted of patients with pulmonary symptoms. These symptoms were determined by an X-ray, CT scan, or MRI revealing bronchitis or terminal bronchitis, tracheitis, or pneumonia, without evidence of other systemic diseases.

The RTI with chronic lung disease group (RTICLDG) consisted of patients with an RTI and a history of asthma, tuberculosis, or chronic fiber disease.

The RTI with severe non-pulmonary symptoms group (RTISNPSG) consisted of patients with severe disorders of the digestive system or nervous system, allergic diseases, idiopathic fever, or convulsions who were also suspected to have an RTI.

The RTI with immunocompromised diseases group (RTIIDG) consisted of RTI patients with lung cancer, leukemia, or HIV and patients who had undergone organ transplantation.

### Real-time polymerase chain reaction (RT-PCR) for pathogen detection

Each sample was simultaneously tested for the following 18 pathogens: IAV, IBV, PIV (1, 2, 3 and 4), RSV, ADV, HRV, HMPV, HCoV (229E, OC43, NL63, and HKU1), HBoV, enterovirus (EV), *Mycoplasma pneumoniae* (MP) and *Chlamydia pneumoniae* (CP). HRV was tested for beginning on July 1^st^ 2011. Detailed protocols for PCR detection have been described in previous reports [9–11].

### Statistical analysis

The statistical analysis was performed using SPSS 19.0. The χ2 test and Fisher’s exact test were used where appropriate. All of the tests were two tailed, and a value of *P* < 0.05 represented statistical significance.

## Results

### General characteristics of distributions

**Fig 1:**
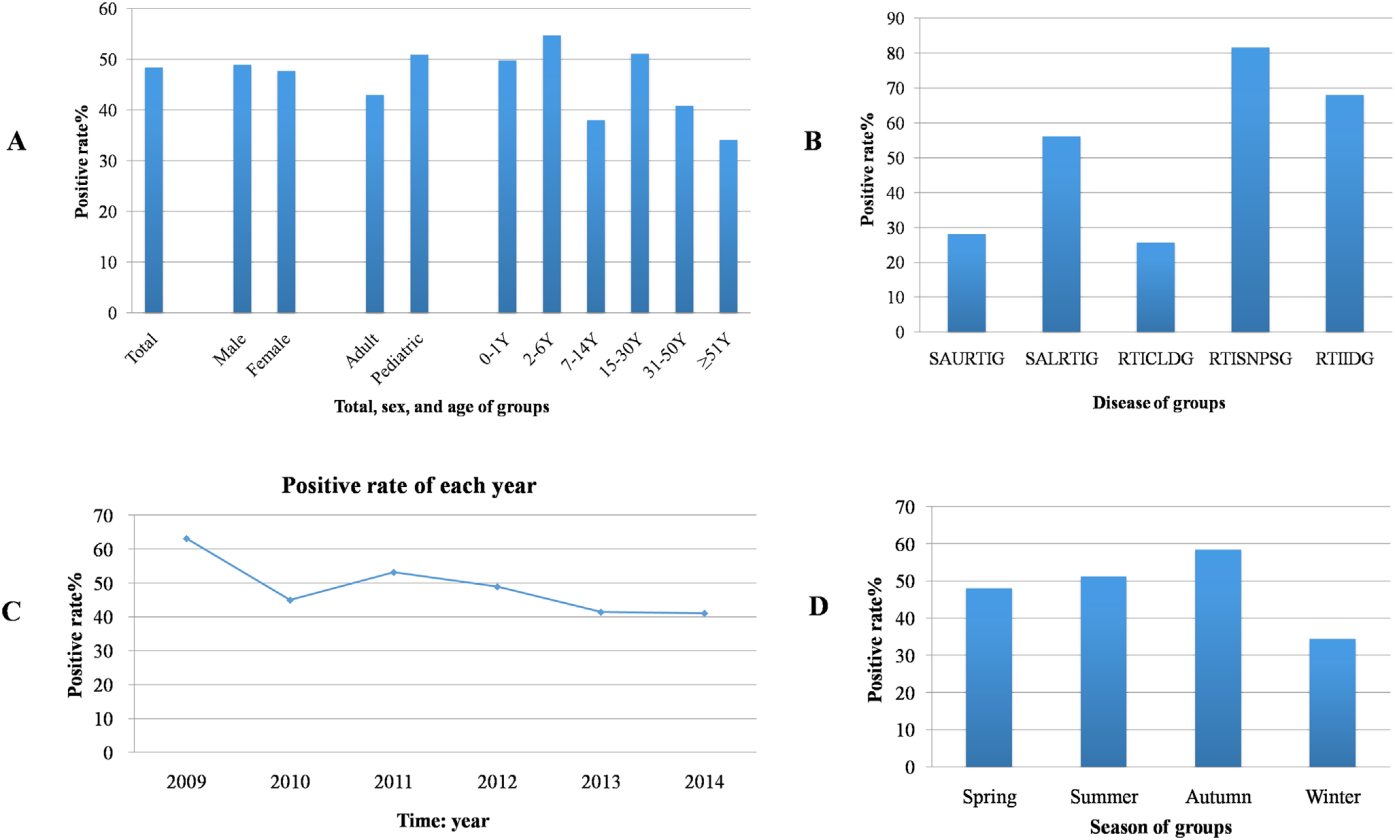
The overall distribution of the pathogens from July 2009 to June 2014. A, the positive detection rates (PDRs) according to patient total, sex, age and its subgroups; B, the PDRs of 5 different disease subgroups. (1) SAURTIG: The single acute upper RTI group. (2) SALRTIG: The single acute lower RTI group. (3) RTICLDG: The RTI with chronic lung disease group. (4) RTISNPSG: The RTI with severe non-pulmonary symptoms group. (5) RTIIDG: The RTI with immunocompromised diseases group; C, yearly PDRs; D, seasonal PDRs.

The overall PDR was 48.42% (6,054/12,502 samples). The PDR in female (47.68%), was lower than that among males (48.92%); however, this difference was not statistically significant. The PDR in pediatric patients was 50.93%, which was significantly higher than the PDR in adult patients (43.00%). The 0–1 and 2-6-year old subgroups showed higher PDRs than the 7-14-year old subgroup did (*P* < 0.05), and the 15–30 and 31-50-year old subgroups showed higher PDRs than the subgroup composed of individuals ≥ 51years old did (*P* < 0.05). The specific PDRs of these subgroups were 49.79%, 54.70%, 38.02%, 51.16%, 40.65%, and 34.19%, respectively (Fig 1A and Table 1).

**Table 1:** The total PDRs and CIRs of respiratory pathogens from July 2009 to June 2014.

Groups	Total(n)	Total positive rate%(n_1_/n)	*P*_*1*_Value	Co-infection rate%(n_2_/n_1_)	*P*_*2*_Value
**Total**	12502	48.42(6054/12502)		17.99(1089/6054)	
**Male**	7523	48.92(3680/7523)	0.176	22.75(837/3680)	<0.05
**Female**	4979	47.68(2374/4979)		10.62(252/2374)	
**Pediatric**	8523	50.93(4341/8523)	<0.05	22.00(955/4341)	<0.05
**Adult**	3979	43.00(1711/3979)		7.83(134/1711)	
**0-1Y**	3591	49.79(1788/3591)	<0.05	21.09(377/1788)	<0.05
**2-6Y**	4064	54.70(2223/4064)		18.00(400/2223)	
**7-14Y**	868	38.02(330/868)		53.94(178/330)	
**15-30Y**	1634	51.16(836/1634)		6.34(53/836)	
**31-50Y**	1134	40.83(463/1134)		9.29(43/463)	
**≥51Y**	1211	34.19(414/1211)		9.18(38/414)	
**SAURTIG**	3586	28.08(1007/3586)	<0.05	15.99(161/1007)	<0.05
**SALRTIG**	4507	56.07(2527/4507)		16.66(421/2527)	
**RTICLDG**	1841	25.69(473/1841)		35.3(167/473)	
**RTISNPSG**	2212	81.60(1805/2212)		15.4(278/1805)	
**RTIIDG**	356	67.98(242/356)		28.1(68/242)	

n: Represents the total number of samples in 5-years.

n_1_: Represents the total number of positive samples in 5-years.

n_2_: Represents the total number of co-infected samples in 5-years.

*P*_*1*_: Represents the *P* values comparing gender, age, five disease groups for the total PDR respectively.

*P*_*2*_: Represents the *P* values comparing gender, age, five disease groups for the total CIR respectively.

Y: Represents year’s old.

(1) SAURTIG: The single acute upper RTI group. (2) SALRTIG: The single acute lower RTI group. (3) RTICLDG: The RTI with chronic lung disease group. (4) RTISNPSG: The RTI with severe non-pulmonary symptoms group. (5) RTIIDG: The RTI with immunocompromised diseases group.

Clinically, the PDRs in the SAURTIG, SALRTIG, RTICLDG, RTISNPSG, and RTIIDG were 28.08%, 56.07%, 25.69%, 81.6%, and 67.98%, respectively, showing that the PDRs in the RTISNPSG, SALRTIG and RTIIDG were higher than the PDRs in the SAURTIG and RTICLDG (*P*< 0.05). However, we observed no statistically significant difference in PDR between the SAURTIG and RTICLDG or between any possible pair among the SALRTIG, RTISNPSG, and RTIIDG (Fig 1B and Table 1). Over the five years from 2009–2014, the total PDR gradually decreased (Fig 1C). In contrast with our previous findings [12], the total PDR in pediatric patients decreased over only the final three years of the analysis. Notably, during the initial three years of the analysis, from 2009–2012, the PDR in pediatric patients was 55.7% (2,361/4,242), which was higher than the PDR from 2009–2014 of 50.93% (4,341/8,524) (*P*< 0.05) (Fig 1A).

Seasonally, the PDRs in summer and autumn were higher than those in winter and spring (*P*< 0.05). The specific seasonal PDRs were: 48.01% (1,668/3,474) in spring, 51.12% (1,646/3,216) in summer, 58.43% (1,806/3,091) in autumn, and 34.33% (934/2,721) in winter (Fig 1D).

### Overall epidemic tendencies

**Fig 2:**
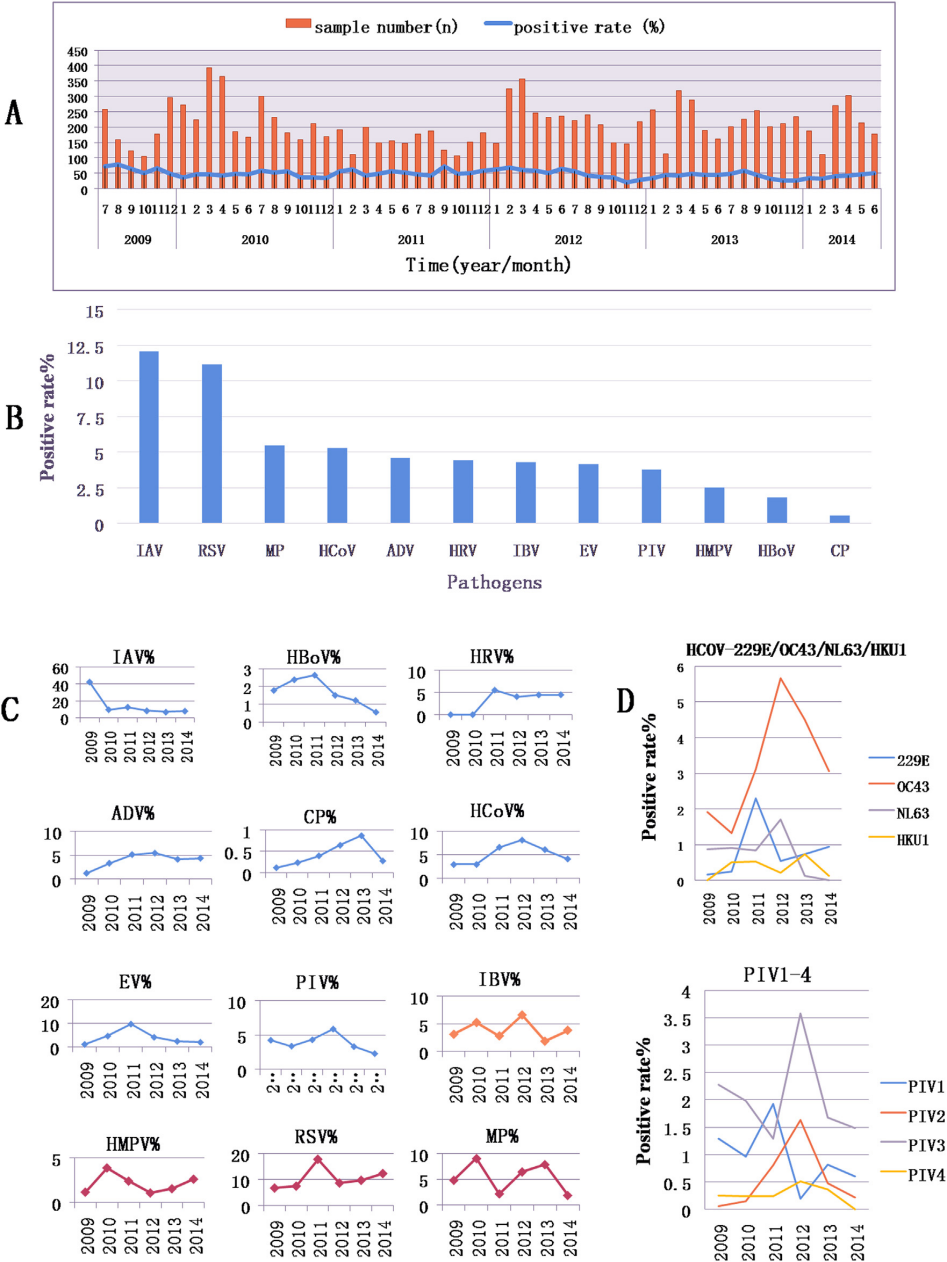
Pathogens’ prevalence over 5 years. A, the total number of samples and the PDR patterns in each month of each year; B, the prevalence patterns of different pathogens over five years; **C**, prevalence peaks over 5 years: one peak (lines in blue), two peaks (lines in red), and three peaks (lines in orange), (%):typical indicates the positive rate of pathogens; D, the yearly prevalence patterns of the 4 types of HCoV and 4 types of PIV.

More RTI patients visited a doctor between March and May than during the other months. The rate of doctor visits was particularly low in January, February, September and October (Fig 2A). However, the total PDR of the pathogens during these months was not significantly different from that in the other months. The 18 pathogens were detected as follows, in decreasing order of PDR: IAV, RSV, MP, and HCoV (the order of the PDRs of the four HCoV types was OC43, 229E, NL63, HKU1), all of which had PDRs greater than 5%, followed by ADV, HRV, IBV, EV, PIV (the order of the four PIV types was PIV3, PIV1, PIV2, PIV4), HMPV, HBoV, and CP (Fig 2B and 2D). During the 5-year period of the study (2009–2014), the individual PDRs of the 18 pathogens exhibited four distinct trends (Fig 2C and 2D): (1) One peak in PDR was observed for IAV (which had a peak in 2009 mainly driven by an outbreak), HBoV, HRV, ADV, CP, HCoV, EV, and PIV. Among these pathogens, a peak-valley trend was observed for IAV, HBoV, and HRV (first tested for on July 1^st^, 2011); a valley-peak trend was observed for ADV, CP, and HCoV (OC43 and 229E); and a valley-peak-valley trend was observed for EV, HCoV (NL63), and PIV2. (2) Two peaks in PDR were observed for HMPV, RSV, and MP. (3) Three peaks in PDR were observed for IBV. (4) No peaks in PDR were observed for HKU1 and PIV4.

### The pattern of pathogen’s PDR depends on the season of detection and on patient sex and age

#### Four distinct seasonal distributions and 2 epidemic tendencies

**Fig 3:**
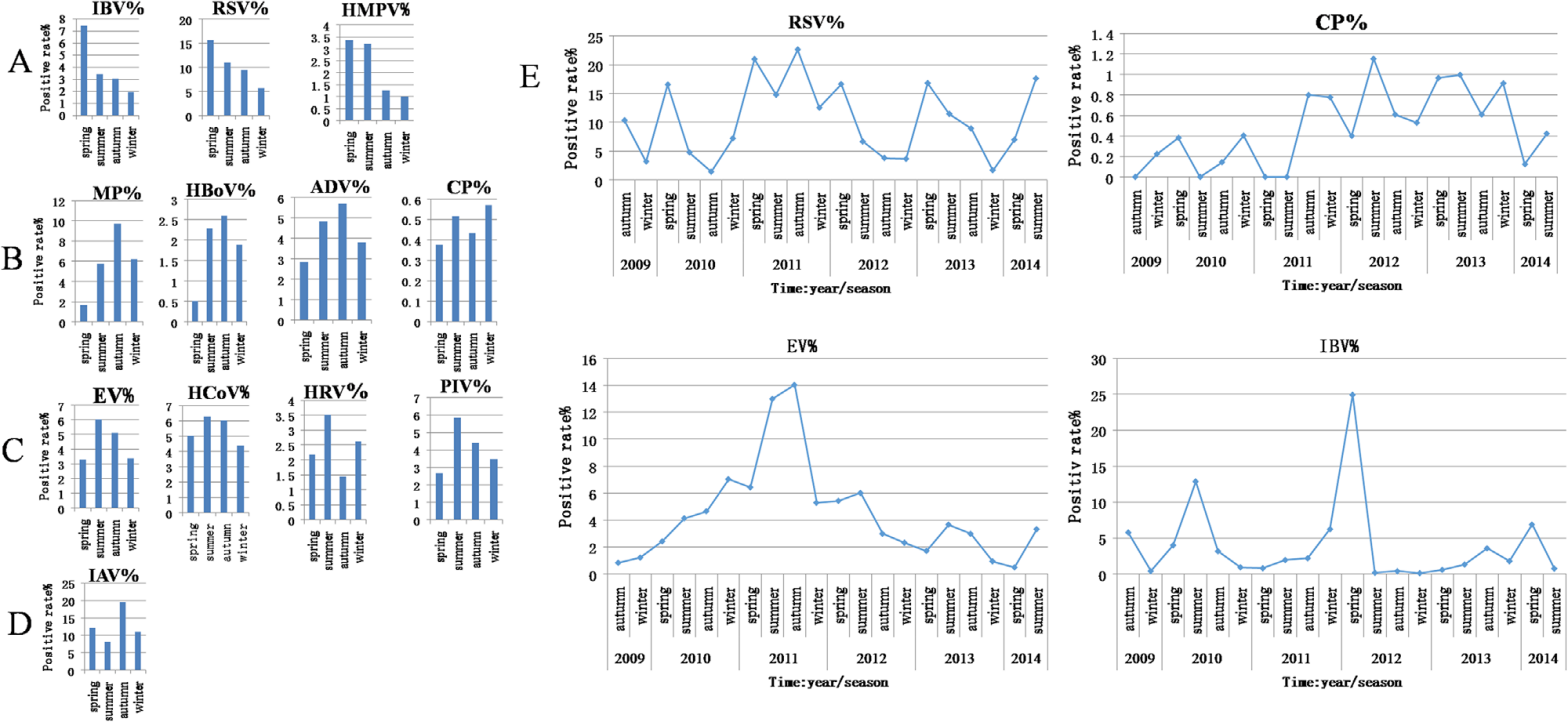
The seasonal prevalence patterns over five years. A, B, C, and D: the seasonal PDRs among 12 kinds of pathogens; E, contains the regular (upper row) and irregular (bottom row) seasonal PDR patterns between July 2009 and June 2014.

IBV, RSV, and HMPV displayed a PDR that gradually decreased as the year progressed from spring onward (Fig 3A). MP, HBoV, ADV, and CP were detected with a lower PDR in the spring than in summer, autumn or winter (Fig 3B). IAV had a peak PDR in autumn and a nadir in summer (Fig 3D). EV, HCoV, HRV, and PIV showed a peak PDR in summer (Fig 3C). Over the course of this study, the individual PDRs of the 18 pathogens exhibited 2 types of epidemic tendency based on seasonal changes in their incidence (Fig 3E). Pathogens that showed a seasonal epidemic peak every year included RSV, CP, IAV, HCoV, PIV, HBoV, HRV, ADV, and HMPV, and the pathogens that did not show a yearly seasonal peak included EV (in children), IBV, CP (in adults), HBoV, HKU1, and PIV4. Overall, season-dependent trends resulted in a lower PDR in winter and spring and a higher PDR in the summer and autumn.

#### Distribution of pathogens according to patient sex

We observed 12 pathogens that showed significant differences in incidence according to patient sex. Specifically, male patients across all ages and risk groups displayed higher PDRs for RSV, PIV (PIV1, PIV3, and PIV4), HRV and CP than female patients did. In contrast, female patients displayed higher PDRs for IBV, MP, HMPV, HCoV-229E, and ADV (Table 2).

**Table 2:** The prevalence of different respiratory pathogens from July 2009 to June 2014.

Pathogen	Male (n = 7523)	Female(n = 4979)	*P* ^*$*^ Value	SAURTIG (n = 3586)	SALRTIG (n = 4507)	RTICLDG (n = 1841)	RTISNPSG (n = 2212)	RTIIDG (n = 356)	P*Value
**IAV**	844(11.22)	545(10.95)	0.634	437(12.19)	270(5.99)	116(6.3)	699(31.6^)^ ^#^	85(23.88)	<0.05
**IBV**	278(3.70)	223(4.48)	<0.05	73(2.04)	79(1.75)	28(1.52)	174(7.87)^#^	16(4.49)	<0.05
**RSV**	956(12.71)	421(8.56)	<0.05	78(2.18)	837(18.57)^#^	78(4.24)	281(12.7)	52(14.61)	<0.05
**MP**	369(4.91)	298(5.99)	<0.05	126(3.15)	324(7.19)	51(2.77)	117(5.29)	39(10.96)^#^	<0.05
**HMPV**	199(2.65)	103(2.69)	<0.05	54(1.51)	120(2.62)	26(1.41)	85(3.84)^#^	5(1.4)	<0.05
**HCoV**	359(4.77)	223(4.48)	0.446	103(2.87)	172(3.81)	42(2.28)	232(10.49)^#^	37(10.39)	<0.05
229E	41(0.54)	45(0.9)	<0.05	8(0.22)	37(0.82)	5(0.27)	33(1.49)	8(2.25)^#^	<0.05
OC43	242(3.22)	141(2.83)	0.221	79(2.2)	115(2.55)	25(1.36)	154(6.96)^#^	24(6.74)	<0.05
NL63	47(0.62)	24(0.48)	0.229	2(0.06)	11(0.24)	6(0.33)	41(1.85)^#^	2(0.56)	<0.05
HKU1	29(0.39)	13(0.26)	0.239	14(0.39)	9(0.2)	6(0.33)	4(0.18)	3(0.84)^#^	0.12
**PIV**	311(4.13)	130(2.61)	<0.05	66(1.84)	198(4.39)	17(0.92)	71(3.21)	17(4.78)^#^	<0.05
PVI1	73(0.97)	27(0.54)	<0.05	9(0.25)	39(0.86)	6(0.32)	11(0.49)	5(1.4)^#^	<0.05
PIV2	49(0.65)	25(0.5)	0.287	17(0.47)	24(0.53)	0(0)	21(0.95)^#^	0(0)	<0.05
PIV3	154(2.05)	77(1.55)	<0.05	37(1.03)	125(2.73)	8(0.43)	29(1.31)	10(2.81)^#^	<0.05
PIV4	35(0.47)	1(0.02)	<0.05	3(0.08)	10(0.22)	3(0.16)	10(0.45)	2(0.56)^#^	<0.05
**EV**	306(4.06)	178(3.6)	0.162	36(1)	153(3.39)	32(1.74)	152(6.87)^#^	22(6.18)	<0.05
**HBoV**	144(1.91)	79(1.58)	0.176	23(0.64)	86(1.9)	18(0.98)	24(1.08)	13(3.65)^#^	<0.05
**HRV**	252(3.35)	91(1.83)	<0.05	72(2)	122(2.71)	59(3.2)^#^	55(2.49)	4(1.12)	<0.05
**CP**	47(0.62)	18(0.36)	<0.05	14(0.39)	27(0.6)	5(0.27)	14(0.63)^#^	1(0.28)	0.298
**ADV**	304(4.04)	254(5.1)	<0.05	70(1.95)	216(4.79)	62(3.38)	85(3.84)	30(8.43)^#^	<0.05

No. (**%**) of each group except where specifically stated.

***P*** ^***$***^: Represents the P values comparing male and female groups.

P*: Represents the P values comparing the five disease groups: (1) SAURTIG: The single acute upper RTI group. (2) SALRTIG: The single acute lower RTI group. (3) RTICLDG: The RTI with chronic lung disease group. (4) RTISNPSG: The RTI with severe non-pulmonary symptoms group. (5) RTIIDG: The RTI with immunocompromised disease group.

^#^: The pathogen with the highest PDR.

#### Distribution of pathogens in adult and pediatric patients

**Fig 4:**
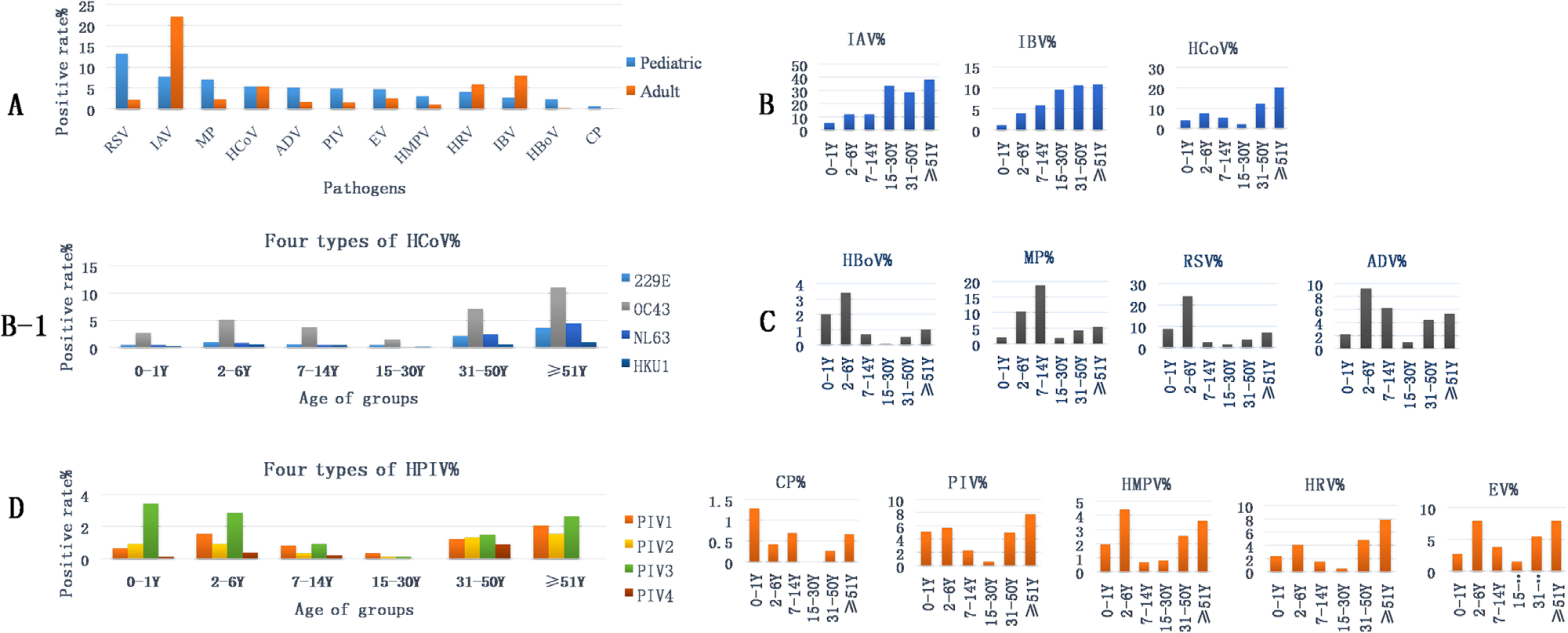
Pathogens’ distributions within age subgroups over five years. A, PDR differences in pediatric and adults subgroups; the (%) in B, B-1, C, D, and E indicates the PDRs of pathogens in different age subgroups.

In pediatric patients, the order of the individual PDRs of the 18 pathogens from highest to lowest was RSV, IAV, MP, HCoV (the order of the PDRs of the four HCoV types was OC43, NL63, 229 E, HKU1), ADV (> 5%), PIV (the order of the PDRs of the four PIV types was PIV3, PIV1, PIV2, PIV4), EV (close to 5%), HRV, HMPV, IBV, HBoV, and CP. In adult patients, the order of the PDRs of the pathogens from highest to lowest was: IAV, IBV, HCoV (the order of the PDRs of the four HCoV types was OC43, 229E, NL63, HKU1), HRV (> 5%), EV, MP, RSV, ADV, PIV (the order of the PDRs of the four PIV types was PIV2 PIV3, PIV1, PIV4), HMPV, HBoV, and CP. The PDRs of 7 of the pathogens (RSV, ADV, MP, HBoV, HMPV, PIV3, and CP) were higher in pediatric patients than in adult patients (*P*< 0.05). However, the PDRs of 7 other pathogens (IAV, IBV, HCoV-229E/OC43/NL63, PIV4, and HRV) were lower in pediatric patients than in adult patients (*P* < 0.05). The remaining 4 pathogens (EV, HKU1, PIV1, and PIV2) were detected at similar rates in both the adult and pediatric patients (*P*> 0.05) (Fig 4A, 4(B-1) and 4D). Three distinct trends in PDR were observed in the age subgroups and were used to divide the pathogens into three types. Type 1 pathogens included IAV, IBV, and HCoV, which exhibited increasing PDRs as patient age increased (Fig 4B). Type 2 pathogens HBoV, MP, RSV, and ADV, which showed increased PDRs during childhood, decreased PDRs during adulthood, and increased PDRs during older age (Fig 4C). CP, PIV, HMPV, HRV, and EV were Type 3 pathogens and displayed lower PDRs in the middle-age subgroups than in the younger and older-age subgroups. PIV4 was not observed in the 15-30-year old subgroup or in the ≥ 51years old subgroup (Fig 4D).

### Clinical characteristics of the pathogens

#### Disease and pathogen distributions in vulnerable populations

In the SAURTIG, the PDR of IAV was greater than 5%. In the SALRTIG, the PDRs of IAV, RSV and MP were greater than 5%, and the PDR of RSV was higher in this group than in the other groups (*P*< 0.05). In the RTICLDG, the PDR of IAV was higher than 5%, and the PDR of HRV was higher in this group than in any other group (*P*< 0.05). Furthermore, PIV2 was not observed in this group. In the RTISNPSG, the PDRs of IAV, IBV, RSV, MP, EV and OC43 exceeded 5%. Moreover, the PDRs of IAV, IBV, HMPV, EV, CP, OC43, NL63, and HCoV were higher in the RTISNPSG than in the other groups (*P*< 0.05). In the RTIIDG, the PDRs of IAV, RSV, MP, OC43, ADV and EV were greater than 5%. The PDRs of MP, HBoV, ADV, and PIV were higher in the RTIIDG than in the other groups (*P*< 0.05) (Table 2).

#### Characteristics of co-infections

**Fig 5:**
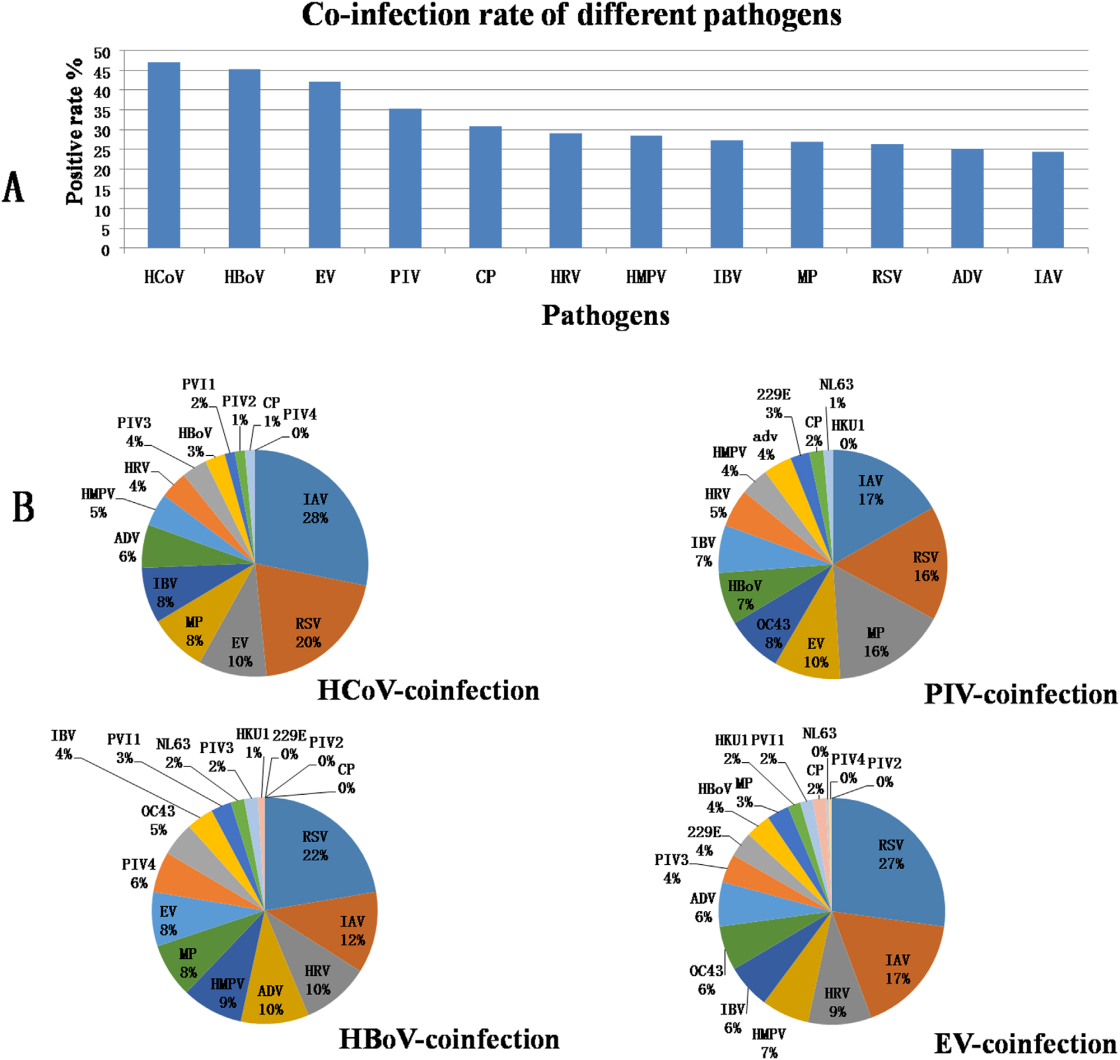
The distribution of co-infections over five years. A, CIRs for different pathogens; B, the 4 highest pathogen CIRs and their constituent ratios.

The overall co-infection rate (CIR) was 17.99%. The male CIR was higher than the female CIR. After stratification based on age, the CIR in pediatric patients was higher than the adult CIR. In pediatric patients, the CIR in the 7–14 year old subgroup was higher than in any other age group, whereas no significant differences in CIR were observed among the age subgroups of adult patients. In addition, excluding RSV, all of the pathogens showed higher CIRs in the pediatric patients than in the adult patients. In children aged 7–14, HCoV-229E was a co-infection in all of the positive samples (5/5), a pattern which was also observed for NL63 (1/1) and PIV2 (2/2). HBoV (1/1) co-infected one patient in the 15-30-year old subgroup. In contrast, HBoV (0/4) and PIV2 (0/1) did not show co-infection in the ≥ 51 years old subgroup. In the groups stratified based on disease, the CIRs of the RTICLDG and RTIIDG were higher than those of the other three groups (*P*< 0.05). However, no significant difference in CIR was observed between any possible pair among the SAURTIG, SALRTIG and RTISNPSG (Table 1). NL63 (2/2) and HKU1 (3/3) appeared to co-infect all of the HKU1-positive samples in the RTIIDG; these two pathogens were also observed to co-infect the CP-positive samples (5/5) in the RTICLDG. However, PIV2-positive samples (0/2) did not show co-infection in the RTIIDG; this pattern was similarly observed for PIV4 positive samples in the SAURTIG (0/3) and RTIIDG (0/2).PIV2 (0/0) was not observed in the RTICLDG, and CP (0/14) was not observed in the RTISNPSG. The four pathogens with the highest CIRs were HCoV (47.07%, 313/665), HBoV (45.18%, 103/228), EV (42.18%, 221/524), and PIV (35.23%, 167/474). In contrast, the lowest CIRs were observed for RSV, ADV and IAV. The incidence of co-infections was clearly different from the rate of single-pathogen infections (Fig 5A). The co-infection combinations for the 4 pathogens with the highest CIR values listed from highest to lowest were as follows: HCoV co-infected with IAV, RSV, EV, MP, IBV, or ADV; HBoV co-infected with RSV, IAV, HRV, ADV, HMPV, or MP; EV co-infected with RSV, IAV, HRV, HMPV, IBV or HCoV-OC43; and PIV co-infected with IAV, RSV, MP, EV, HCoV-OC43 or HBoV (Fig 5B).

## Discussion and Conclusion

In developed countries, respiratory pathogens, such as viruses, MP, and CP, have been extensively researched [12, 13]. However, we conducted the present study because limited research on these pathogens has been performed in developing countries. For this purpose, we conducted the present study.

Our results show that, although the number of patients with RTIs differed in each month, the PDRs were similar throughout the study period, indicating that the composition of these respiratory pathogens in the environment is stable. Among the pathogens tested, IAV, RSV, MP, and HCoV were detected with PDRs greater than 5%. These results are consistent with the high prevalence of these viruses, especially IAV and RSV, which remain the greatest threats to the human respiratory system [14]. Additionally RSV, MP, HBoV, HRV and ADV exhibited increasing incidences in children over the course of the study. During the same period, in some developed countries such as the USA, European countries, RSV, influenza and HMPV became completely undetectable. HRV has been reported to be one of the main pathogens causing upper RTIs, with a PDR as high as 10–30% [15]. This finding is inconsistent with our research, which observed the PDR of HRV to be less than 5%, indicating it is not a common pathogen in RTIs. This difference may be due to two factors. First, we tested pharyngeal swabs but not nasal swabs. Second, HRV causes common upper RTIs, which often present with symptoms too mild to lead to doctor visits or hospitalizations. RTIs are the most common disease observed in humans and are especially prevalent in pediatric and immunocompromised populations [14]. On a related note, different behavioral habits, environments, and degrees of immunity are experienced at different ages [16]. In addition, because the half-lives of antibodies that recognize different pathogens vary [17, 18], there are differences in disease incidence and severity according to age. Studies [19, 20] have shown that approximately 100% of 6-year old children have been infected with one or more respiratory pathogens; however, these infections do not result in effective long-term immunity, so children can be repeatedly infected by the same viruses. The results of the present study suggested that among adults, attention should be focused on IAV, IBV, HCoV, and HRV infections, whereas in pediatric patients, RSV, MP, IAV, OC43 and ADV infections should receive the most attention. Moreover, IAV, MP, and OC43 infections should be closely monitored by clinical detection throughout childhood, and RSV and ADV infections should be monitored in preschool and school-aged children, respectively.

The overall PDR was not significantly different between male and female patients. However, the incidences of certain pathogens such as RSV, PIV4, and HRV also did differ by gender. To our knowledge, this result has not been previously reported, and the reasons behind this observation should be explored in future studies.

The seasonal climate is an important factor that can affect pathogen transmission [21]. In the current study, the season affected the total PDR, which increased from winter to spring to summer and reached a peak in autumn. The fact that the PDR of the RTIs was higher in summer and autumn is in contrast with the seasonal incidence of RTIs. This seasonal trend is likely due to a more hospitable environment for the viruses, the artificial selection of susceptible human populations, and the method of transmission of the pathogens [21]. Consistent with previous studies, our results found that most pathogens exhibited obvious seasonal epidemic peaks each year. However, IBV, HBoV, HKU1, PIV4, pediatric EV, and adult CP did not show annual seasonal peaks. Surprisingly, the PDR of pediatric EV increased each year until a peak was reached and then an annual decrease in PDR occurred. The reason for this trend is not clear. While the PDR of RSV was highest in spring, the PDRs of the common pathogens IAV, MP, HCoV, EV, and ADV were highest in autumn. Based on these results, the detection of viruses that cause RTIs should be targeted based on the season.

Clinically, respiratory pathogens play an important role in chronic lung disease patients and in immunocompromised patients [22–24]. RTIs can be particularly exacerbated in immunocompromised patients, such as patients with cancer, blood diseases, or an organ transplant, which can result in patient mortality [25–27]. Previous reports have shown that approximately 25–45% of immunocompromised patients die due to an RTI [25]. Novel molecular techniques have enabled the identification of new viruses, such as HMPV, HCoV, HBoV, and HRV, as causative agents of RTIs. A pathogen PDR greater than 5% suggests that the pathogen is common in a disease group, and when a disease group experiences a PDR higher than that in other disease groups, the disease group is particularly vulnerable to the pathogen. In the present study, different disease groups had distinct common pathogens and were vulnerable to different pathogens. RTIs are most often co-infections caused by multiple viruses, multiple bacteria, or a combination of viruses and bacteria [28–30]. These pathogens can interact with each other, resulting in more severe symptoms [31–34]. Although some research [35–37] showed that the CIR ranged from 10% to 46% in pediatric patients, the samples used for detection, population studied and time factors were different in those studies than in the present study. In the current study, common pathogens associated with co-infections were HCoV, HBoV, EV, and PIV. Previous studies have shown that HCoV co-infection does not result in more severe disease [38]. Alternatively, pathogens that co-infected patients positive for HBoV have been reported [19, 39, 40] to cause clinical symptoms ranging from negligible to severe, and co-infection with EV and PIV is often accompanied by severe symptoms that involve other body systems [41, 42]. Immunocompromised patients in particular are susceptible to the four most common co-infectious pathogens. Therefore, immunocompromised populations should be targeted by RTI prevention strategies. Additionally, the highest CIRs were observed in the < 7 year and ≤ 14 year old subgroups in the present study, and the CIRs of the pathogens, except RSV were higher in pediatric patients than in adult patients. The reasons for these findings must be determined by future studies.

Our research represents a large epidemiologic study of RTIs. As mentioned in our previous article [11], bronchial alveolar lavage fluid (BALF) from the site of infection is the best sample for testing for a lower RTI. However, because harvesting BALF is rarely carried out, especially in ill children, we analyzed the characteristics of pathogens from pharyngeal swab samples. Outpatients often have symptoms that are too mild to allow for pathogen detection from such samples, so we chose to use inpatients for this research. In addition, according to the manufacturer’s data, the sensitivities of the detection kits used were as follows: 1000 copies/ml including: influenza A virus (IAV), influenza B virus (IBV), four types of parainfluenza (PIV1, PIV2, PIV3, PIV4), respiratory syncytial virus (RSV), enterovirus (EV), human metapneumovirus (HMPV), four strains of human coronavirus (HCoV-229E, OC43, NL63 and HKU1). And 500 copies/ml including: adenovirus (ADV), human bocavirus (HBoV), Mycoplasma pneumoniae (MP), Chlamydophila pneumoniae (CP), and human rhinovirus (HRV). In summary, based on the pathogen distributions and epidemic tendencies, our results showed that IAV remains the most common RTI pathogen, as IAV exhibits a strong propensity for pathogenic mutations, increased awareness regarding IAV is warranted. Overall, summer and autumn appear to be the key periods for RTI-associated viral pathogen prevention and surveillance. Notably, spring is the most important time for the specific prevention of RSV, and EV should be monitored in pediatric patients year-round. More importantly, pathogens such as RSV, ADV, HCoV, HRV, and CP have exhibited increasing incidences in recent years, which suggests the need to strengthen prevention and monitoring measures, especially for severe pathogens such as RSV. Our results also suggest that children require different preventative measures than adults due to differences in pathogen susceptibility. In RTI patients with severe extra pulmonary symptoms, viral pathogens are most likely the cause of the RTI. The populations most susceptible to co-infection include school-aged male children, patients with chronic lung disease, and immunodeficient individuals, and the pathogens most commonly involved in co-infections are HCoV, HBoV, EV, and PIV. In conclusion, despite the complexity of RTIs, the clinical and epidemiological signatures of the disease can provide important clues to aid clinicians in optimizing diagnostic and treatment plans and to enable the generation of reasonable public health strategies. In addition, vaccines and new drug treatments are urgently needed.
